# Smoking habits and benign prostatic hyperplasia: A systematic review and meta-analysis of observational studies

**DOI:** 10.1097/MD.0000000000004565

**Published:** 2016-08-12

**Authors:** Huan Xu, Shi Fu, Yanbo Chen, Qi Chen, Meng Gu, Zhong Wang

**Affiliations:** Department of Urology, Shanghai Ninth People's Hospital, Shanghai Jiaotong University School of Medicine, Shanghai, China.

**Keywords:** benign prostatic hyperplasia, cigarette, meta-analysis, observational, smoking

## Abstract

Previous studies have warned against the promoting effects of cigarette smoking on benign prostatic hyperplasia (BPH). In contrast, some have argued that smoking confers a protective effect regarding BPH, while others have observed an aggravated effect. Thus, we performed this meta-analysis to determine whether cigarette use is associated with BPH risk.

To identify articles from observational studies of relevance, a search was performed concurrent to March 21, 2016, on PubMed, Web of Science, Cochrane, EBSCO, and EMBASE databases. Random-effect model, according to the heterogeneity, was calculated to reveal the relative risks (RRs) and corresponding 95% confidence intervals (CIs).

Eight articles were included in this meta-analysis, representing data for 44,100 subjects, of which 5221 (11.8%) had BPH as defined according to the criteria. Seven reports are concerned with analysis between nonsmokers and ex-smokers, in which no significant difference was observed (RR = 0.99, 95% CI 0.94–1.05). Another meta-analysis of 7 studies indicated an observable trend, but without significant difference between groups of nonsmokers and current smokers (RR = 1.17, 95% CI 0.98–1.41). Between groups of heavy (6 articles; RR = 1.02, 95% CI 0.84–1.24) and light smokers (5 articles; RR = 0.90, 95% CI 0.71–1.15), again no significant difference appears. Finally, we combined individuals as never-smokers and ever-smokers and still found no significant difference between the 2 groups of patients (RR = 1.03, 95% CI 0.92–1.15). Sensitivity analysis was displayed and confirmed the stability of the present results.

Combined evidence from observational studies shows no significant association between cigarette smoking and BPH risk, either for ex-smokers or for current smokers. The trend of elevated BPH risk from smoking was observed only in current smokers compared with nonsmokers, while marginal significance was observed in comparing ever-smokers with never-smokers in operative patients with BPH.

## 1 Introduction

Benign prostatic hyperplasia (BPH) is one of the most common urinary disorders in older males, which condition is histologically characterized by increased tissue mass and hyperplasic cells. As a type of multifactorial disease, there are many pathogenic processes involved in development of BPH, such as chronic inflammation, oxidative stress, hypoxia, and ischemia.[ ^1^ ^2^] As is known, many living habits, together called “lifestyle,” contribute greatly to benign hyperplasia of the prostate. As is accepted by many urologists, the metabolic disease, such as obesity and diabetes, aggravates prostatic hyperplasia. Gacci et al ^[3]^ published a meta-analysis in 2015 that underlines the exacerbating role of metabolic disorders in development of BPH. Otherwise, the association between smoking tobacco (cigarette and/or pipe) and BPH has been investigated in a number of studies with little conclusive evidence regarding this association.

Cigarette smoking is a known risk factor of several disorders, ^[4]^ yet the relationship between smoking and BPH morbidity remains arguable. Some have concluded that smoking aggravates lower urinary tract symptoms (LUTS), while others argue that decreased prostate volume is associated with smoking and have even expounded that cigarette smoking delays the hyperplasia. Confronted with the varied opinion currently in print on causes of BPH, no articles have yet summarized the relevant literature. To estimate the risk of BPH from smoking tobacco, meta-analysis is overdue to examine the associations between available reports.

Furthermore, the main treatments for prostatic enlargement are drug therapy and surgery. Different therapies are used according to the urinary symptoms; therefore identifying any modifiable risk factors for BPH is important, particularly where BPH leads to surgery. Accordingly, we undertook this meta-analysis of observational studies to discuss the relationship between BPH and smoking.

## 2 Materials and methods

### 2.1 Literature search

This meta-analysis was conducted following guidance provided by the Cochrane Handbook ^[5]^ and is reported according to the Preferred Reporting Items for Systematic Reviews and Meta-Analysis Guidelines (PRISMA) ^[6]^ as well as MOOSE guidelines. To identify articles from observational studies updated on March 21, 2016, a full search was performed of relevant publications on PubMed, Web of Science, Cochrane, EBSCO, and EMBASE. Only studies published in English were considered and search terms included: “cigarette,” “smoking,” “tobacco,” “prostatic hyperplasia,” and “prostate enlargement.” In order to expand the searching scope, we expanded keywords such as cigarette smoking or BPH. Additionally, we searched the references of the final selected articles one by one and found no new passages fitting for the entry criteria mentioned in the following.

### 2.2 Study selection

Using the previously described approach, 2 reviewers (HX and SF) independently selected eligible trials and identified a total of 1246 articles. From this list, 749 articles were excluded on the basis of duplication, leaving 497 articles from that set. From these, 470 articles were excluded according to elements of the title or abstract. Finally, 8 studies were fully qualified for this meta-analysis, following the criteria: original research, human studies, observational articles, and providing information about associations between smoking and BPH. Relative risk (RR) estimates are included in this meta-analysis. As BPH is not a rare disease, only those studies were included that provide data required for calculations according to the formula. ^[7]^

### 2.3 Data extraction

Two independent reviewers (HX and SF) collected the data and entered these into a purpose-designed Excel form: name of first author, publishing date, study period, patient source, mean age of participants, study design, study conclusion, number of subjects, smoking criteria, and BPH, which provided detailed data and confounding factors for matching or adjustments. Following this, methodological quality of each report was assessed by the reviewers. Quality Assessment Forms were utilized only for cross-sectional research, while Newcastle–Ottawa Scale (NOS) was applied to cohort studies and case–control studies.

### 2.4 Heterogeneity analysis

To better understand the possible source of heterogeneity among studies, meta-regression analysis was performed. Age, investigated area, control for confounding factors, publication year, study design, and follow-up time were tested for the heterogeneity analysis.

### 2.5 Data synthesis and analysis

Homogeneity of effects across studies was calculated using Cochran Q and quantified according to I^2^ statistics, by which heterogeneity was interpreted as 0% to 40%: might not be important; 30% to 60%: may represent moderate heterogeneity; 50% to 90%: may represent substantial heterogeneity; and 75% to 100%: considerable heterogeneity. ^[5]^ Though smoking histories vary, there are 3 groups in most articles, including current smokers, ex-smokers, and nonsmokers. Here, subgroup meta-analysis is designed according to the articles published by Botteri et al. ^[8]^ Subtitle studies were performed according to smoking history, diagnosis criteria, and amount of smoking. Where heterogeneity was indicated, summary estimates were based on the random-effect model, covering those included studies of varying effect levels. Where included studies had the same effect level,[ ^9^ ^10^] the summary estimate is based on the fixed-effect model. Though some of our studies show low heterogeneity, random-effect model was still presented. First, it was because of the limited number of included studies. Second, the fixed-effect model assumed no variability in the underlying effect across studies while in light of the observations in the other groups, substantial between-study heterogeneity was observed. Finally, we summarized risk estimates, 95% confidence intervals (CIs), and tabulated forest plots. Publication bias was assessed using Begg and Egger tests. Because few studies publish the quantitative details of cigarette use, only light or heavy categories of smoking groups are included for meta-analysis, with 20 cigarettes/d as a dividing line. The analyses were performed using Revman 5.2 (Review Manager 5.2.10, Oracle Corporation, Denmark), while Begg and Egger tests, as well as meta-regression, were obtained using Stata version 14.0 (Stata Corporation, College Station, TX, USA).

### 2.6 Ethical statement

As all analyses were grounded on previous publications, ethical approval was not necessary.

## 3 Results

### 3.1 Study selection and characteristics

**Figure 1:**
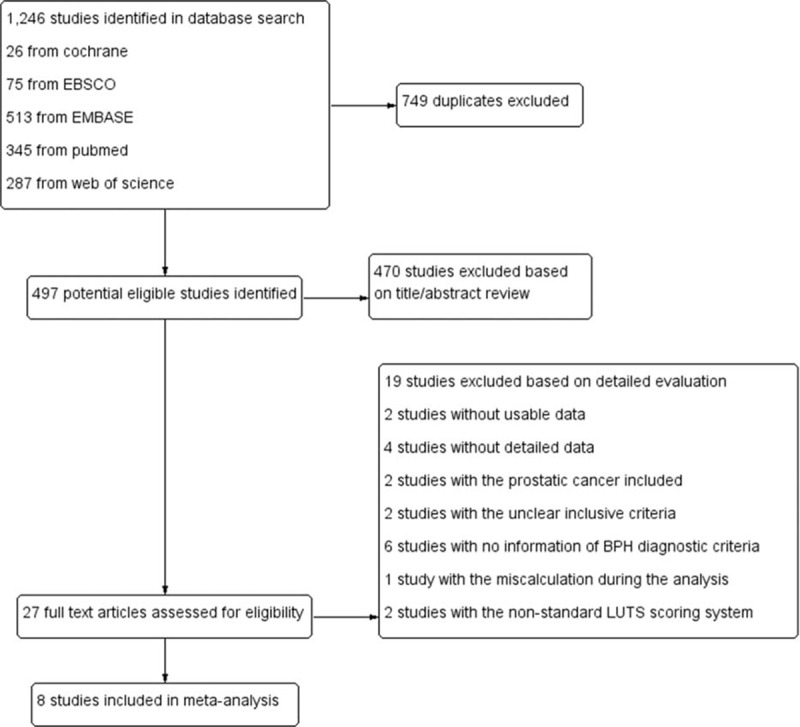
Flow diagram for study selection.

Figure 1 shows the flow diagram for study selection. From database searches, a total of 1246 citations regarding cigarette use and BPH were discovered. From the search set, 749 studies were excluded by criteria. On the basis of titles and abstracts, we identified 27 suitable full-text articles. After detailed evaluation, 19 more studies were excluded: 2 for absence of data on incidence rate of BPH, 4 for lack of detailed data, 2 for inclusion of prostatic cancer, 2 for lack of clear inclusive smoking criteria, 6 for insufficient details on BPH diagnosis criteria, 2 which applied an informal scoring system for LUTS, and 1 that contained a calculation error. Finally, 8 eligible studies [^11^ ^12^ ^13^ ^14^ ^15^ ^16^ ^17^ ^18^] were identified totally, including 6 cohort studies, 1 case–control study, and 1 cross-sectional study. Of the final studies, 5 were conducted in United States, 1 in Europe, 1 was in Australia, and 1 lacks identification of the study area. According to NOS and agency for healthcare research and quality criteria ^[19]^ (shown in Table 1), all included studies were deemed to be of high quality.

**Table 1:**
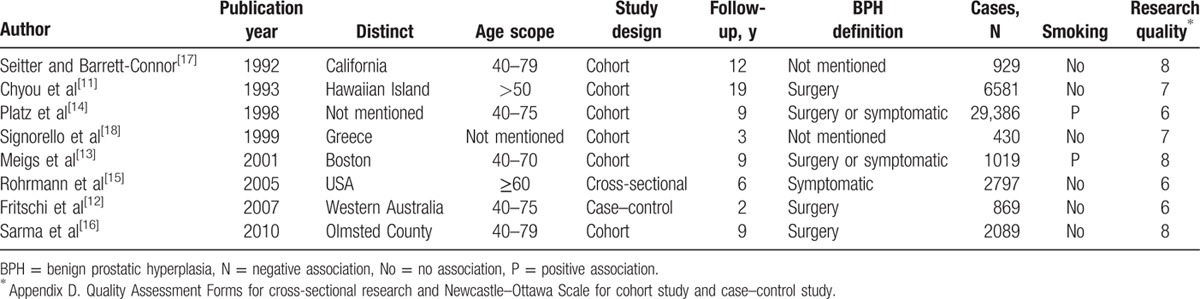
Studies in this meta-analysis.

### 3.2 Meta-analysis results

#### 3.2.1 Nonsmokers vs ex-smokers

Though heterogeneity (I^2^ = 6%, *P* = 0.38) was low in this group, the random-effect mode was still chosen to provide an estimate of RR and its 95% CI. In this comparison, no associated risk was found between the 2 groups (RR = 0.99, 95% CI 0.94–1.05, Fig. 2 A). Among these articles, BPH criteria differed. Some diagnosis followed symptoms, some followed surgery, and histology. When stratifying studies by diagnosis criteria, no statistically significant association was observed in the surgically diagnosed population (RR = 1.02, 95% CI 0.92–1.13, Fig. 2 B) under random-effects evaluation. No publication bias was observed in the 2 analysis (nonsmokers and ex-smokers: *P*_Begg_ test = 0.230, *P*_Egger_ test = 0.240; surgically diagnosed patients: *P*_Begg_ test = 0.602, *P*_Egger_ test = 0.766).

**Figure 2:**
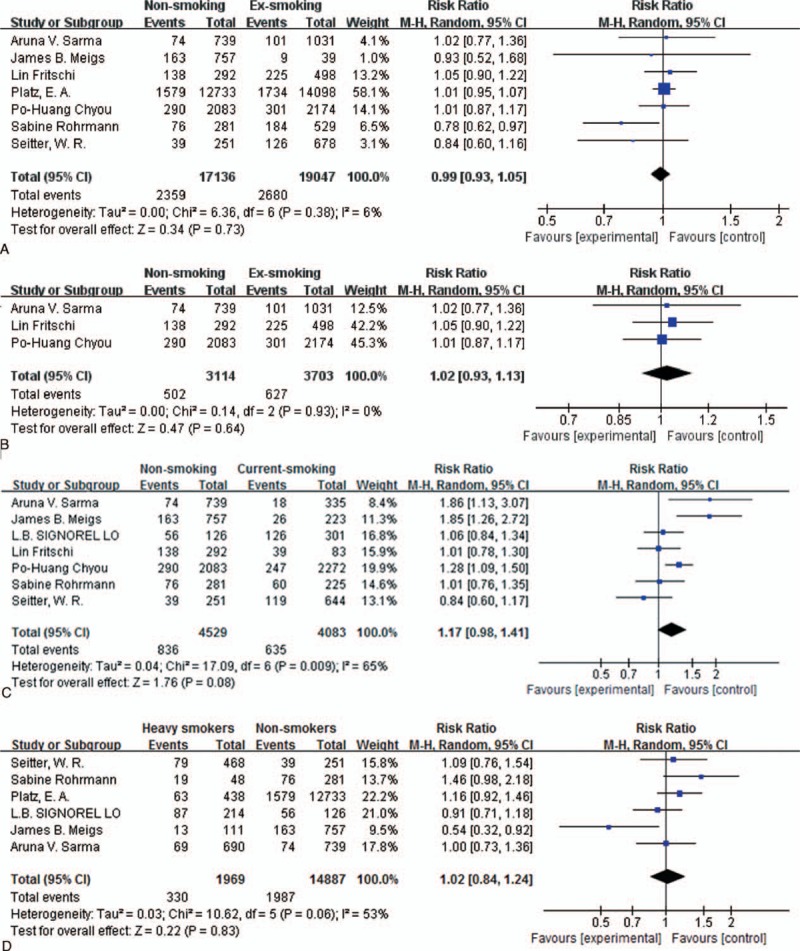
Forest plot: (A) comparison of nonsmokers versus ex-smokers and risk of BPH, (B) comparison of nonsmokers versus ex-smokers and risk of BPH diagnosed with only surgical treatment, (C) comparison of nonsmokers versus current smokers and risk of BPH, (D) comparison of nonsmokers versus current smokers and risk of BPH diagnosed with only surgical treatment, (E) analysis of effect of heavy smoking toward BPH risk, (F) analysis of effect of light smoking toward BPH risk, (G) comparison of never-smokers versus ever-smokers and risk of BPH, (H) comparison of never-smokers versus ever-smokers and risk of BPH diagnosed only with surgery. BPH = benign prostatic hyperplasia.

**Figure 2 (Continued):**
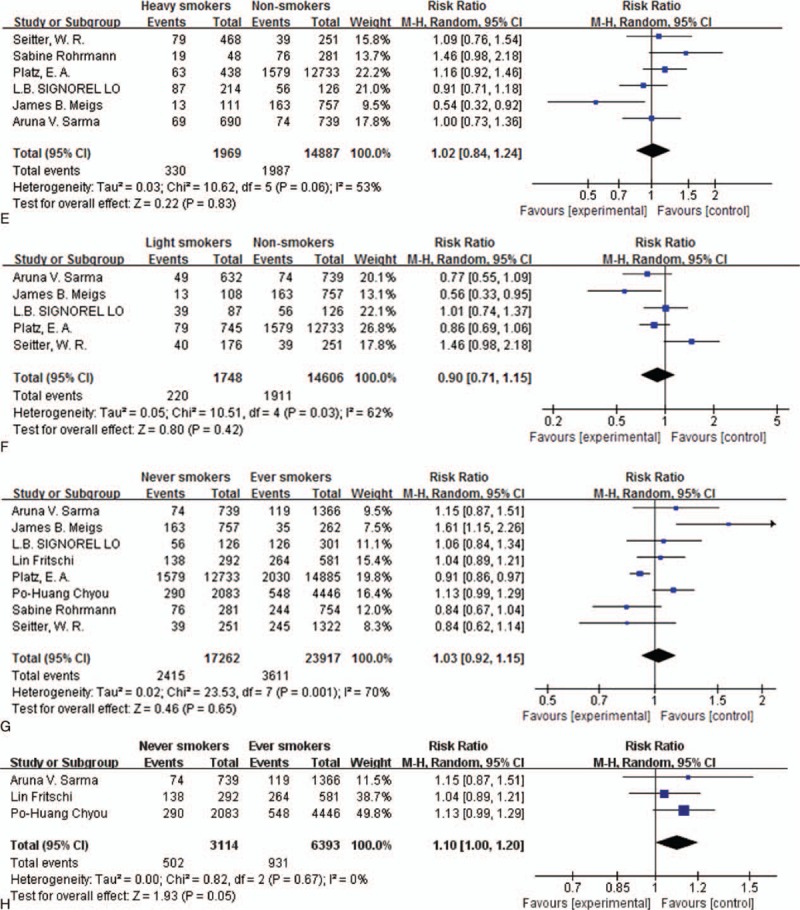
Forest plot: (A) comparison of nonsmokers versus ex-smokers and risk of BPH, (B) comparison of nonsmokers versus ex-smokers and risk of BPH diagnosed with only surgical treatment, (C) comparison of nonsmokers versus current smokers and risk of BPH, (D) comparison of nonsmokers versus current smokers and risk of BPH diagnosed with only surgical treatment, (E) analysis of effect of heavy smoking toward BPH risk, (F) analysis of effect of light smoking toward BPH risk, (G) comparison of never-smokers versus ever-smokers and risk of BPH, (H) comparison of never-smokers versus ever-smokers and risk of BPH diagnosed only with surgery. BPH = benign prostatic hyperplasia.

#### 3.2.2 Nonsmokers vs current smokers

Because substantial heterogeneity (I^2^ = 65%, *P* = 0.009) was observed, we applied the random-effects model and found that smoking is not associated with increased risk of BPH (RR = 1.17, 95% CI 0.98–1.41, Fig. 2 C) in this case. Diagnosis criteria did not affect the pooled results (RR = 1.26, 95% CI 0.97–1.63, Fig. 2 D), while heterogeneity (I^2^ = 63%, *P* = 0.07) was analyzed by the random-effects model rather than by the fixed-effects model. After omission of the article of Meigs et al, heterogeneity decreased with no changes to results. In the publication analysis, no bias was observed in the group of nonsmokers versus current smokers (*P*_Begg_ test = 0.881, *P*_Egger_ test = 0.858) and the studies of surgical treated BPH (*P*_Begg_ test = 0.602, *P*_Egger_ test = 0.813).

#### 3.2.3 Nonsmokers vs heavy and light smokers

In order to study the potential dose-dependent effect of cigarette smoking on BPH, we divided subjects into heavy and light smokers according to their habits, with a division at 20 cigarettes/d. In the heavy smokers group, heterogeneity (I^2^ = 53%, *P* = 0.06) was observed, and under the selected random-effects mode, no significant difference was observed between the 2 groups (RR = 1.02, 95% CI 0.84–1.24, Fig. 2 E). In the light smokers group, similar heterogeneity existed (I^2^ = 62%, *P* = 0.03), while analysis still found no difference (RR = 0.90, 95% CI 0.71–1.15, Fig. 2 F). Meigs et al's report was the main source of heterogeneity, which did not affect the final result by our analysis. When investigating nonsmokers versus heavy smokers and risk of BPH, no publication bias was observed (*P*_Begg_ test = 0.851, *P*_Egger_ test = 0.387). No publication bias was observed as well in the investigation of light smokers (*P*_Begg_ test = 0.624, *P*_Egger_ test = 0.998).

#### 3.2.4 Never-smokers vs ever-smokers

Some selected articles report 3 groups (ex-smokers, current smokers, and nonsmokers) in studies on effects of smoking, while some publications include only 2 groups (smokers and nonsmokers) for final analysis. Thus, we combined ex-smokers and current smokers in performance of the meta-analysis. In the combined group, no significance appeared when comparing never-smokers and ever-smokers (RR = 1.03, 95% CI 0.92–1.15, Fig. 2 G). Regarding the surgical group, no heterogeneity (I^2^ = 0%, *P* = 0.67) was observed and only marginal significance (RR = 1.11, 95% CI 1.01–1.22, Fig. 2 H) was found. No publication bias was observed investigating never-smokers versus ever-smokers and risk of BPH (*P*_Begg_ test = 0.322, *P*_Egger_ test = 0.156). In the analysis of surgical treated group, none of the bias was present (*P*_Begg_ test = 0.602, *P*_Egger_ test = 0.961).

#### 3.2.5 Publication bias and heterogeneity analysis

Publication bias was measured through Begg and Egger tests (Stata 14.0, Stata Corporation), in which no significant publication bias was observed by group analysis. For the heterogeneity analysis, age, investigated area, control for confounding factors, publication year, study design, and follow-up time, which may be potential sources of heterogeneity, were tested. However, none of the above factors was responsible for the observed inter-study heterogeneity. We next eliminated studies one by one as sources of heterogeneity, and found that the study by Meigs et al was the main divergence point in all group comparisons. Furthermore, we found removal of this article did not change the final result.

## 4 Discussion

The present study indicates that cigarette smoking is associated neither with increased nor decreased BPH morbidity, except for the existence of light significant difference in surgical patients with BPH when comparing “never-smokers versus ever-smokers” groups.

BPH is characterized by nonmalignant enlargement of the prostate, as well as by enrichment of epithelial and stromal cells, which is prevalent in older men. As has been reported previously, over 60% of men aged more than 50 years old are troubled by BPH, while the rate increases to 80% after 70 years. ^[20]^ As a kind of multifactorial disease, BPH may be caused by different factors, including chronic inflammation, oxidative stress, hypoxia, ischemia, and so on.[ ^1^ ^2^ ^21^] All of the above can contribute to accumulation of prostatic cells. Overwhelming and widely accepted evidence concludes that smoking causes local hypoxia, oxidative stress, endothelial injury, and chronic inflammation of various organs. [^22^ ^23^ ^24^ ^25^] There still remain disputes, however, in the study of the relationship between smoking habit and BPH.

Some investigators report a moderate inverse association between smoking and BPH when detected by symptoms or clinical examination.[ ^11^ ^15^ ^26^ ^27^ ^28^] Some studies even report a protective effect by smoking against BPH. ^[29]^ By contrast, others argue that cigarette smoking accelerates the morbidity of BPH. Yet again, some urologists conclude that the effect of smoking tobacco on BPH turns on the amount smoked.[ ^14^ ^30^] There has been no meta-analysis, however, studying the relationship between smoking and BPH. In this meta-analysis, we combined 8 original articles, published before March 21, 2016, all of which are observational studies, consisting of 6 cohort studies, 1 cross-sectional study, and 1 case–control study. As well, the effects of different smoking histories, smoking amount, and entry criteria are studied here. Study type was not analyzed as an independent subgroup because only 2 included studies are not cohort studies. Results here indicate no statistically significant association between cigarette use and risk of BPH. In order to compare smoking's effects on different groups of patients with BPH, individuals were grouped as “never-smokers versus current smokers,” “never-smokers versus ex-smokers,” “light/heavy smokers versus nonsmokers,” and “never-smokers versus ever-smokers.” Among the listed comparison groups, no effect of smoking on development of BPH appears statistically. To make the BPH diagnosis more precise, studies using symptomatic criteria were excluded, though their exclusion yields only light advantage for smokers, in the “never-smokers versus ever-smokers” group (*P* = 0.05), when undergoing BPH surgery.

It has been accepted that plasma steroid hormone levels are affected by cigarette smoking. Higher testosterone levels were observed for smokers in previous studies, which tends to be associated with higher intraprostatic dihydrotestosterone (DHT) levels, a key factor for BPH.[ ^15^ ^31^] Nicotine in cigarettes has been shown to lead to increased DTH level in the prostate and increased sympathetic nervous system activity, contributing greatly to BPH and LUTS.[ ^15^ ^32^] Moreover, previous publications have proved that the serum pH caused by cigarette and tobacco use also plays an important role in decreasing serum zinc levels, affecting the amount of both testosterone and DHT in the prostate ^[33]^ and participating in the development of BPH pathology. Furthermore, smoking injures blood vessels to a degree, which encourages the enlargement process, as poor circulation is a factor in BPH. In spite of the existing basic evidence, our meta-analysis does not indicate the resulting effects’ presence in BPH, while the negative effect of smoking may truly influence the perioperative performance in BPH or the advent of LUTS. Furthermore, only 2 articles present the data for prostatic volume in their study[ ^16^ ^30^] and none of them shows a significant difference in prostatic volumes of smokers compared with never-smokers. Adversely, Plaz et al present their conclusion that smoking decreases prostate enlargement to a degree and even argue for a protective effect of smoking against prostate enlargement. ^[14]^ In addition, Sarma et al reported smoking decreases acute urine retention significantly, though this has little relation with BPH. In fact, some other studies have observed that smoking decreases acute postoperative urine retention rate. ^[16]^ In this way, smoking elevates the bladder activity through sympathetic nervous system and may potentially aggravate over active bladder symptoms. It is also probably because of the autonomic muscle relaxation caused by nicotine with the first sphincter being autonomic. Thus, the meta-analysis of cigarette smoking's effect on LUTS needs further study.

Limitations of this meta-analysis should also be noted. Individual patient or original data were not available limiting our ability to do more detailed analyses. In our search process, there are 2 studies of large sample population[ ^34^ ^35^] that did not, however, present usable data for our analysis and were excluded. As well, insufficient follow-up durations could have affected our conclusions about the smoking and BPH mortality. Although the research analyzed is of worldwide origin, data are lacking from Asian and African countries. In the included reports, no distinction between pipe/cigar tobacco and cigarette was performed; thus, different types of smoking were not analyzed as subgroups. Furthermore, our results may not be valid enough to extrapolate all BPH populations and may not have sufficient data to assess the risk as a small number of studies were used. With limited articles having been carried out, we are also looking forward for more studies about this aspect in further studies in the future.

Heterogeneity in some groups is significant and worth noting. First, observed heterogeneity may be due to differences in chosen criteria for smoking. Second, the diagnostic method for BPH varies. For BPH, the gold standard is histologic analysis, and few studies met this standard. Third, though most studies separate individuals into smokers and nonsmokers, the detailed duration of smoking habit for each patient is unclear. In the analysis of the heterogeneity, the report by Meigs et al may be the main source. As we analyzed, the following may be the reasons. First, some patients smoked pipes or cigars and the authors classified them as nonsmokers because classifying them as current smokers or noncigarette smokers did not affect the results. Second, the authors used interviews to the patients to defined the systematic BPH: Do you have frequent urination? Do you have difficulty urinating? and Have you ever been told by a health professional that you have an enlarged or swollen prostate? In the other reports using systematic BPH criteria, the authors used symptom index from American Urological Association. Moreover, with the varied levels of medical conditions at that time, the equipment and technologies are different. The author used “Have you ever been told by a health professional that you have an enlarged or swollen prostate?” may be biased in this way. However, we deleted this report in our analysis and found no change in the final result. Thus, we reserved this article in this meta-analysis.

In conclusion, our results suggest that there may be no significant association between smoking and risk of BPH. Strong evidence remains lacking for increased risk of BPH surgery among smokers, including ex-smokers and current smokers, though a marginally significant difference was observed in ever-smokers when compared with never-smokers. More studies are needed to detail the effects of smoking on risk of BPH.
